# Multi-field-sensing metasurface with robust self-adaptive reconfigurability

**DOI:** 10.1515/nanoph-2023-0050

**Published:** 2023-03-02

**Authors:** Ruichao Zhu, Jiafu Wang, Chang Ding, Yajuan Han, Yuxiang Jia, Sai Sui, Tianshuo Qiu, Zuntian Chu, Hongya Chen, Jun Wang, Bo Feng, Shaobo Qu

**Affiliations:** Shaanxi Key Laboratory of Artificially-Structured Functional Materials and Devices, Air Force Engineering University, Xi’an, Shaanxi 710051, China

**Keywords:** adaptively reconfigurable metasurface, intellectualization, multi-beam, multispectral detection

## Abstract

The continuous increase in communication capacity is accompanied by an increase in transmission frequency, which creates new demands on the transmission efficiency in modern. Signal relay transmission can increase the transmission distance, however, conventional repeaters relay the signal in a specified direction, which is difficult to accommodate communication when a receiving device suddenly appears around the repeater. In this work, we propose a new signal transmission repeater, which is implemented by an adaptively reconfigurable multi-beam reflective metasurface based on multispectral detection. The reconfigurable metasurface with varactor diodes is designed and the mapping of phase profiles to voltages is established by polynomial fitting method. Visual, laser, infrared and ultrasonic detectors are used to detect targets in different scenarios. Thus, the detection information is fed back to the reconfigurable metasurface for adaptively multi-beam switching. As verification, the adaptive metasurface repeater was fabricated and measured to verify our design. All the results exhibit consistency with theoretical design. Importantly, this work paves a new way to intelligent metasurfaces and may find applications in intelligent communications, smart home, etc.

## 1 Introduction

Metasurfaces, as two-dimensional metamaterials, realize a series of artificial composite structures with exotic electromagnetic (EM) properties through specific permutations [1]. Metasurface can realize flexible and effective modulation of EM wave polarization, amplitude, phase, polarization mode, propagation mode and other characteristics, which provides unprecedented freedom for manipulating EM waves [2, 3]. Since the metasurface has a high degree of freedom in the manipulation of EM waves, the elaborate design of the metasurface can realize the anomalous refraction [4, 5], polarization conversion [6, 7], EM absorption [8, 9], beam modulation [10, 11], and other functions. Due to its exotic properties, metasurfaces are widely used in antenna technology [12], information processing [13], optical devices [14], etc.

Metasurfaces greatly enhance the freedom of EM wave design and derive a variety of fascinating functions. Multifunctional metasurface is possible through the design of structural units. Conventional metasurfaces are called static metasurfaces as soon as they are designed and their functionality is solidified [1, 15]. Limited by the inherent space, the static metasurface can only show the predetermined functional characteristics. Conversely, the metasurface that can change the functional representation in real time is usually defined as the dynamic metasurface [16–18]. Currently, the metasurface can be reconfigured by mechanical, optical, thermal, and electrical methods to achieve adjustable functionalities [19–22]. In 2014, programming metasurface was proposed to employ electronic components such as diodes as part of a meta-atom to switch functions by changing the feeding mode [23]. Digital metasurface can effectively improve the reconfiguration of metasurface through electronic circuits, which has attracted the attention of many researchers. The electronically adjusted method realizes the modulation of the equivalent parameters of the meta-atom through the design of the structure and the electronic circuit, which in turn realizes the dynamic switching of amplitude [24], phase [25], and polarization [26]. Moreover, programming metasurfaces reach many significantly distinct functions in real time by giving instructions to the field programmable gate array (FPGA) [23, 27, 28]. With the application of FPGA and other microprocessors, the programming metasurface gradually presents the trend of intelligence. For example, the intelligence of the metasurface can be improved by combining the metasurface microwave sensing with intelligent algorithms [29, 30]. Moreover, with the combination of sensors and other perceptrons, the metasurfaces exhibit adaptability to the environment [31–35]. The above works greatly enrich the metasurface design and accelerate the process of metasurface intelligence. The rapid development of communication technologies, such as 5 G/6 G communication, provides a new platform for the application of metasurfaces. The beam modulation by the metasurface can achieve directional communication to the target, which will effectively improve the communication efficiency.

**Figure 1:**
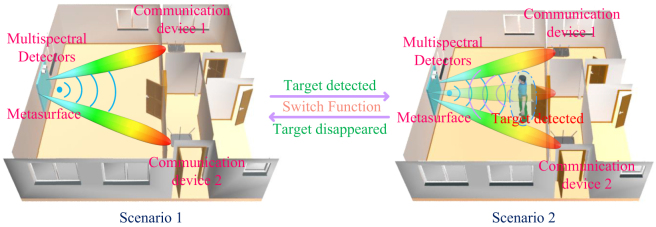
Schematic diagram of multispectral detection empowered adaptively reconfigurable multi-beam reflective metasurface.

In this work, we propose an adaptively reconfigurable multi-beam reflective metasurface system based on multispectral detection technology. The position of the target object is calibrated using ultrasonic detection, infrared detection, laser detection, and visual detection, respectively. Once the object is detected, detector feeds back to the metasurface for beam switching. The reconfigurable metasurface embedded with diodes can modulate the phase response by adjusting the voltage of diodes. The mapping relationship between the phase response and voltage is established by polynomial fitting. With the detector and microprogrammed control unit (MCU) to realize target detection, the EM response of the metasurface is controlled in real time, which can realize adaptive functional modulation according to the changes of environment. Figure 1 illustrates the schematic diagram of this work and conceives an application scenario of modifying the signal repeater, in which the metasurface as signal repeater can switch the EM transmission mode in real time. The metasurface system is suspended on the wall. Under normal circumstances, the metasurface acts as a repeater to relay information to surrounding devices. When a target is detected in the normal direction, the metasurface system adaptively adjusts the voltage to switch the reflected phase, so as to switch the beam scattering mode. At this time, a new beam is extended in the normal direction to ensure the communication of the newly detected object. As verification, this metasurface system is fabricated and measured in microwave anechoic chamber. The theory, simulation and measurement show that the design is effective. This metasurface system continues the framework of sensors-metasurfaces and further extends the metasurface application scenario by applying multispectral detectors. This work paves a new way for metasurface-assisted signal transmission, which will lay the foundation for future intelligent communications.

## 2 Adaptively reconfigurable reflective metasurface

### 2.1 Controllable meta-atom design

**Figure 2:**
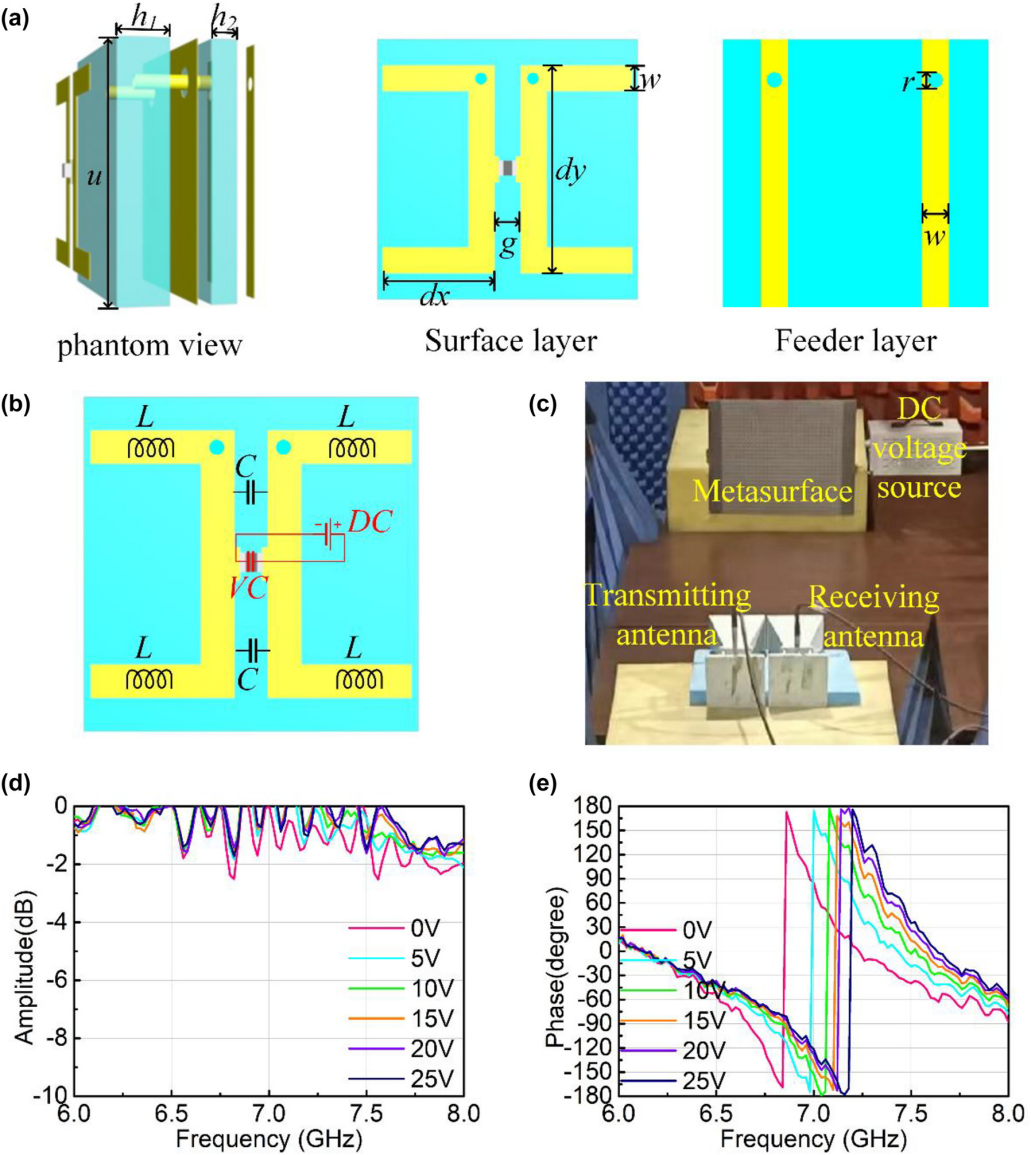
Coding meta-atom with PIN diode: (a) structure and geometrical parameters of the meta-atom; (b) equivalent circuits of the meta-atom; (c) S-parameters measured environment; (d) measured reflectivity response at different voltages; and (e) measured phase response at different voltages.

Here, a frame structure with varactor diodes is designed as the meta-atom for modulating phase response. The geometrical parameters of the structure are shown in Figure 2(a), including the structure layer, feeding layer, substrate, and bias line. The vertical wire length of the metal frame is *dy* = 8 mm, the horizontal wire length is *dx* = 4.3 mm, the width of the metal wire is *w* = 1 mm, and the gap between the two metal frames is *g* = 1 mm. The period size of the meta-atom is *u* = 10 mm. The metal material is copper with electrical conductivity is 5.96 × 10^7^ S/m. The thickness of copper is 0.017 mm. All the metal structures are etched on F4B dielectric substrate with a complex permittivity 2.65(1 + 0.001*j*). The meta-atom consists of multiple layers and the thickness of two F4B dielectric substrates is *h*_1_ = 2 mm and *h*_2_ = 1 mm, respectively. The surface structure layer is separated from the back feeding layer by a metal plate in the middle, which can avoid the influence of the feeding network on EM response. The top and bottom layers are connected via metallized holes with the diameter is *r* = 0.6 mm. The varactor diode is embedded between the two frames and the diode is SMV1405-040LF from Skyworks. The SMV1405 of silicon abrupt junction varactor diodes is designed for use in voltage controlled oscillators (VCOs) requiring tight capacitance tolerances, which has been widely applied in metasurface design [36–39]. The equivalent circuit of this structure is shown in Figure 2(b), where the adjacent metal frames can be equivalent to capacitors and the long metal wires can be equivalent to inductors. Voltage regulation of R-L-C resonance can be realized by introducing varactor diodes in the middle of capacitors. The free space method is employed to measure the S-parameter of the metasurface under different voltages, in which the measurement environment is shown in Figure 2(c). The EM response of the metasurface is modulated by switching different voltages, and the amplitude and phase results of measurement are shown in Figure 2(d) and (e). Figure 2(d) shows the amplitude responses at different voltages, where the amplitude is higher than −1 dB at 7.2 GHZ. Amplitude measurement indicates that the structure has high reflection efficiency. Figure 2(e) illustrates the phase response at different voltages. When the voltage changes from 0 V to 25 V, the phase changes from 0° to 180° at 7.2 GHz. Based on the free modulation of voltage to phase, the EM response of the metasurface can be controlled by the MCU.

### 2.2 Phase customized by voltage polynomial fitting

**Figure 3:**
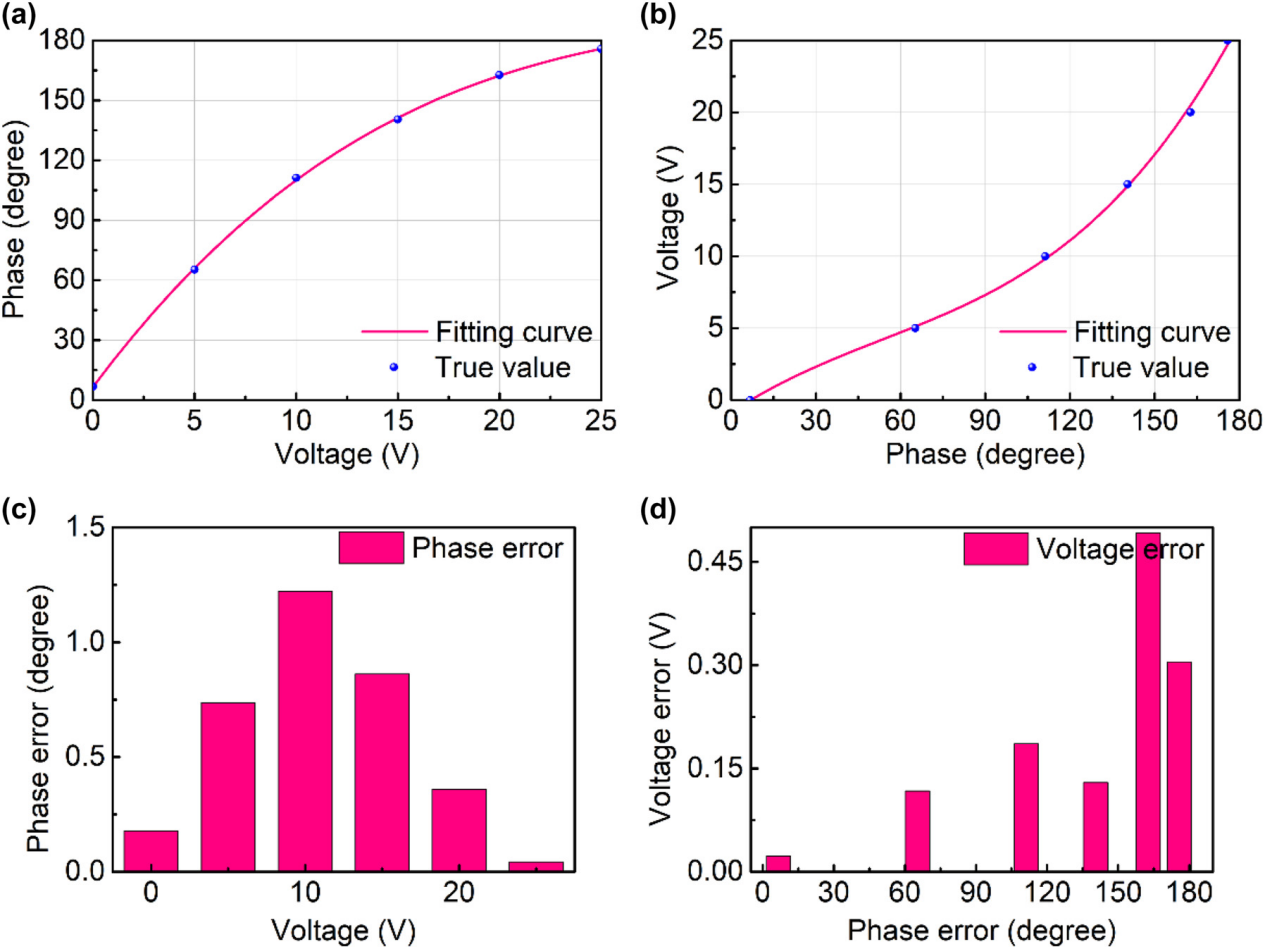
Bidirectional fitting of phase and voltage: (a) forward fitting from voltage to phase; (b) inverse fitting from phase to voltage; (c) phase error between the fitting and true value; and (d) voltage error between the fitting and true value.

According to the relationship between the EM response of the meta-atom and the voltage, phase modulation can be achieved by biased voltages. The continuous voltage variation between 0 and 25 V corresponds to the continuous variation of 0–180° reflection phase, which indicates an intrinsic mapping relationship between phase and voltage. Informative data of voltage-phase are collected and the mapping shows a nonlinear relationship. Therefore, the cubic function is applied to fit the data, and the fitting function is expressed in Equation (1).

(1) $$f\left(x\right)=ax^{3}+bx^{2}+cx+d$$

where *f*(*x*) is the fitting curve function, [*a*, *b*, *c*, *d*] is the coefficient matrix of the function *f*(*x*). Due to the mapping relationship between voltage and phase, the data can be fitted by forward and inverse, respectively. Voltage to phase is defined as a forward design. In forward design, voltage is set to input and phase is set to output. After fitting, the parameters of cubic function [*a*, *b*, *c*, *d*] is [0.00345, −0.3595, 13.6, 6.552], where the fitting performance is shown in Figure 3(a). Oppositely, the mapping from phase to voltage is also fitted by a cubic polynomial and the parameters [*a*, *b*, *c*, *d*] is [0.000006, −0.000976, 0.1293, −0.8508]. The fitting performance of inverse process is shown in Figure 3(b). Through the above data fitting, the bidirectional design between voltage and phase can be realized respectively. R-Square function is used to evaluate the fitting degree of this mapping, which is expressed by Equation (2).

(2) $$R^{2}=\frac{\overset{n}{\underset{i=1}{\sum }}{\left(\hat{y}-\bar{y}\right)}^{2}}{\overset{n}{\underset{i=1}{\sum }}{\left(y_{i}-\bar{y}\right)}^{2}}$$

where *y*_*i*_ is true value, $\bar{y}$ is mean value, $\hat{y}$ is fitting value, *n* is dimension of data. After verification, the fitting degree of forward design is 99.99%, and that of inverse design is 99.91%. The absolute error between the fitting value and true value is shown in Figure 3(c) and (d), which represents the forward error and the inverse error respectively. In forward design, the fitting error of phase is less than 1.5°. In inverse design, the fitting error of voltage is less than 0.5 V. In summary, the phase modulated metasurface can be customized with the mapping between the phase and voltage.

### 2.3 Multispectral detection empowered beam modulation

The continuous increase in communication capacity is accompanied by an increase in transmission frequency, creating new demands on transmission efficiency. With the expansion of communication capacity, there will be loss in high-frequency signal transmission in the air, which leads to the reduction of transmission distance. One solution is to apply signal relay transmission which can increase the transmission distance. However, conventional repeaters relay the signal in a specified direction, which is difficult to accommodate when a receiving device suddenly appears around the repeater. The coding metasurface provides a new method to manipulate EM wave transmission via adjusting the coding sequence. Therefore, metasurface can be applied to relay transmissions. With multispectral detector, the metasurface can realize adaptive scattering mode control. Here, we demonstrate an adaptively reconfigurable multi-beam reflection metasurface system, which is based on multispectral detectors. The multispectral detectors are placed in the normal direction of the metasurface, and the detectors can judge whether there is an object or not, so as to regulate the reflected beam scattering mode.

**Figure 4:**
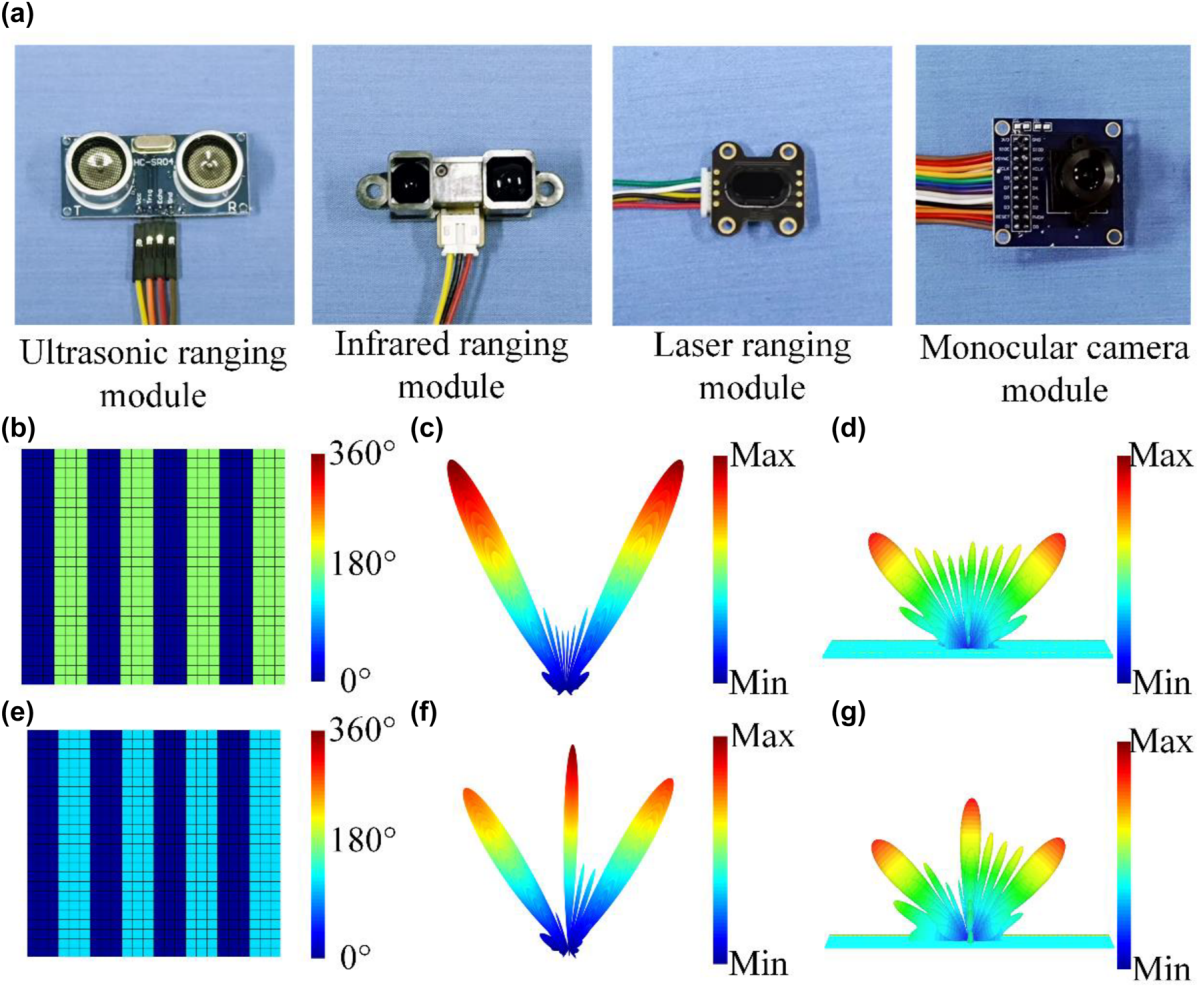
Beam modulation of the metasurface: (a) the four different spectrum detectors that ultrasonic ranging module, infrared ranging module, laser ranging module, and monocular camera module (b) phase profile with 180° phase difference; (c) theoretically calculated far-field beam mode in phase profile at (b); (d) simulated far-field mode in phase profile at (b); (e) phase profile with 120° phase difference; (f) theoretically calculated far-field beam mode in phase profile at (e); (g) simulated far-field mode in phase profile at (e).

In order to extend application scenarios of the adaptive metasurface system, a variety of detectors with different spectra are used to assist the transmission of EM waves. As shown in Figure 4(a), ultrasonic detection, infrared detection, laser detection and visual detection are used to calibrate the position of the target object. The technical parameters of these modules, such as model, maximum range, working voltage, working frequency/wavelength and packaging size, are supplemented in Table 1. The integration of multiple sensors for detection will further expand the application scenarios of metasurfaces, such as ultrasonic modules that are immune to dust or high humidity, infrared that can detect at night, lasers that can provide greater accuracy, and cameras that can provide wider viewing angles. When there is no object, the 2-beam scattering mode is adopted to realize the function of signal repeater. When the target is detected, the 3-beam scattering mode is adopted to radiate the beam normally while realizing the signal repeater.

**Table 1:** The technical parameters of spectrum detectors.

Device	Maximum range	Working voltage	Working frequency/wavelength	Packaging size
Ultrasonic ranging module (HC-SR04)	450 cm	5 V	40 kHz	45 × 20 × 15 mm
Infrared ranging module (GP2Y0A02)	150 cm	4.5–5.5 V	870 nm	30 × 13 × 22 mm
Laser ranging module (TOF200F)	200 cm	3–5 V	0.91 μm	17 × 18 × 6 mm
Monocular camera module (OV7725)	1000 cm	3–5 V	440–760 nm	34 × 35 × 25 mm

According to coding theory, when plane waves impinge on a reflective metasurface, the far-field scattering pattern under different coding sequences can be expressed as [23, 40, 41]:

(3) $$\begin{array}{l} \begin{array}{cc} F\left(\theta ,\varphi \right) & =f_{m,n}\left(\theta ,\varphi \right)\overset{M}{\underset{m=1}{\sum }}\overset{N}{\underset{n=1}{\sum }}\exp\left\{j\left[\varphi_{m,n}\right.\right.\\ & \;+k_{0}D_{x}\left(m-1/2\right)\left(\sin\theta \cos\varphi -\sin\theta_{i}\cos\varphi_{i}\right)\\ & \;\left.\left.+k_{0}D_{y}\left(n-1/2\right)\left(\sin\theta \sin\varphi -\sin\theta_{i}\sin\varphi_{i}\right)\right]\right\} \end{array} \end{array}$$

In which *θ* is elevation angle, *φ* is azimuth angles, *f*_*m*,*n*_(*θ*,*φ*) is the pattern function of the coding unit, which can be regarded as a constant. *k*_*0*_ is the wave vector in free space, *M* and *N* the number of meta-atoms in horizontal and vertical directions, respectively. *Φ*_*m,n*_ the reflected phase at position (*m,n*). *D*_*x*_ and *D*_*y*_ are the sizes of elements in *x* and *y* directions, respectively. *θ*_*i*_ and *φ*_*i*_ is incident angle of incident wave. The deflection angle can be regulated by adjusting the sequence of coding units. In conventional coding metasurface, the element phase difference is 180°. Ideally, the normal beam of the reflected beam cancels out and the reflected beam is scattered to other angles. However, when the phase difference is less than 180°, the main lobe of the reflected wave cannot complete cancellation, and a main lobe will appear in the normal direction.

According to the fitting method mentioned above, the voltage value corresponding to arbitrary phase can be quickly obtained, and the scattered beam can be regulated. Here, the 2-beam and 3-beam scattering modes are demonstrated as the functional verification of the sample. The theoretical phase profiles of 2-beam and 3-beam are shown in Figure 4(b) and (e), in which the meta-atoms with phase 0°, 120°, and 180° are required. After cubic function fitting, it can be fast calculated that the voltage value at this time should be 0 V, 11 V, and 25 V. According to Equation (3), the theoretical scattering beam mode is calculated, and the far-field scattering beam plotted theoretically is shown in Figure 4(c) and (f). Furthermore, CST microwave studio is employed as simulation software to simulate the metasurface in the two predetermined states. The metasurface is placed on *xoy* plane. The boundaries simulation in *x*, *y*, *z* are set to ‘open add space’ to simulate the free space. The *x*-polarized wave is normal incident from *z* direction. Far-filed monitor at 7.2 GHz is added to observe the far-field pattern. Figure 4(d) illustrates the 3D far-field scattering pattern of the metasurface when the phase profile is distributed according to Figure 4(b). Figure 4(g) illustrates the 3D far-field scattering pattern of the metasurface when the phase profile is distributed according to Figure 4(e). The simulated results are basically consistent with the theoretical calculation results, which theoretically validates our design.

## 3 Experiment and test

**Figure 5:**
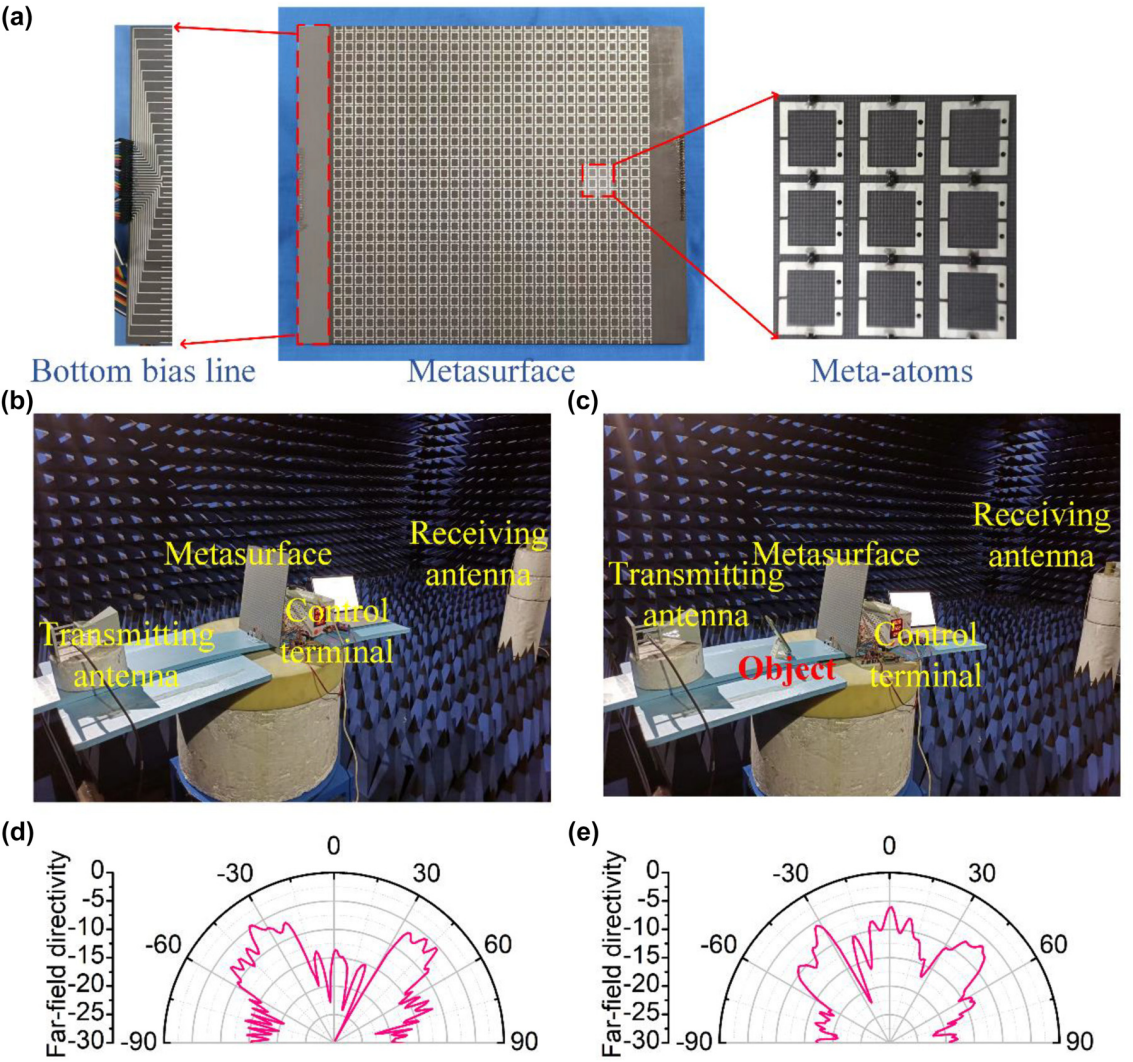
Sample demonstration and measurement verification of the metasurface: (a) photograph of fabricated metasurface prototype (b) far-field measurement environment in microwave anechoic chamber without object (c) far-field measurement environment in microwave anechoic chamber with object (d) the cross profile of far-field measured results of the scenario where object is no here (e) the cross profile of far-field measured results of scenario where object is here.

In order to further verify the adaptively reconfigurable multi-beam reflective metasurface, a prototype of programming metasurface was fabricated and measured, and some detectors were mounted on the system. The metasurface consisting of multiple layers was fabricated by commercial Printed Circuit Board (PCB) techniques and the bias line network is achieved by metallized via holes. The middle layer contains a layer of metal plate through which the feeder passes to realize the power supply design, which is to achieve the isolation of the feeding network from the structural pattern. The variable capacitance diode is SMV1405-040LF, which is embedded in meta-atom by means of SOD-882 packaging technology. The photograph of fabricated metasurface prototype is shown in Figure 5(a). The environment of far-field measurement system is carried out in microwave anechoic chamber as shown in Figure 5(b). The metasurface is placed on rotating platform. The designed meta-atoms can theoretically be independently controlled. However, the individual control of each unit means more feeders and ports. Therefore, in order to simplify the test, we will parallel single columns to simplify the test. The feeding network is placed at the posterior end of the metasurface. The supply source is a DC voltage source. Sensors detect the presence of objects and transmit signals to the Arduino Uno R3. Arduino as MCU receives the signals and transmits them to computer. The computer switches the output voltage of the DC voltage source through serial communication. Two instances of the case are shown in the Figure 5, where Figure 5(b) is the case without the object and Figure 5(c) is the case with the object. In the case of normal direction with or without object, the far-field patterns are measured, and the corresponding measured results are shown in the Figure 5(d) and (e). The metasurface exhibits two scattering beams when there is no object in the normal direction. Correspondingly, the metasurface exhibits three scattering beams when there is an object in the normal direction. The measured results are consistent well with the theoretical results, which convincingly demonstrate the effectiveness of the multispectral detection empowered adaptively reconfigurable multi-beam reflective metasurface system.

## 4 Conclusions

In this work, we propose the adaptively reconfigurable multi-beam reflective metasurface which is based on multispectral detection technology. The detection sensors of ultrasonic, infrared, laser and vision are employed to detect the target objects, which can adapt to different application scenarios. The reconfigurable metasurface consists of an array of meta-atoms embedded with varactor diodes, which can modulate the reflected phase response by adjusting voltage of variode. The mapping between phase and voltage is realized by cubic function fitting, so that arbitrary phase response can be customized to tailor phase modulation. The metasurface can be combined with multispectral detection technology and processor terminal to form an adaptively reconfigurable multi-beam reflective metasurface system. The metasurface system can control the beam scattering mode according to target detection. As an example, we fabricated the prototype of the metasurface system and demonstrated this system in anechoic chamber. All the measured results exhibited the effectiveness of this work. The framework of this metasurface system provides an adaptive application scenario based on multiple sensors. And the capabilities of the metasurface system can be further expanded by integrating more powerful detection equipment. More importantly, this work paves a new way to intelligent metasurfaces and can be widely extended in the fields of intelligent communication and intelligent detection.
